# Facilitators and Challenges of Integrating Experts by Experience Activity in Mental Health Services: Experiences from Finland

**DOI:** 10.1007/s10597-022-01039-0

**Published:** 2022-11-08

**Authors:** Mari Kivistö, Marjatta Martin, Sanna Hautala, Kari Soronen

**Affiliations:** Faculty of Social Sciences, University of Lapland, Rovaniemi, Finland

**Keywords:** Experts by experience, EbyE, Mental health services, Integration, Finland

## Abstract

This qualitative study examines the facilitators and challenges of integrating Experts by Experience (EbyE) activity in mental health services in the public sector from the perspective of mental health professionals and trained EbyE. The research data consist of four focus group interviews conducted in one hospital district in Finland. In the hospital district, EbyE activity is an established working model in mental health services. The professional focus groups had 9, and the EbyE focus groups had 13 participants. The data were analysed using abductive content analysis. The experiences of professionals and EbyE emphasised the successful integration of EbyE activity. The identified facilitators of integration included an organisational culture that values EbyE activity, facilitating operating structures, and extensive implementation and utilization of EbyE activities. Despite successful integration, certain challenges were described with regard to actors and practices. The findings indicate that the hospital district has overcome many of the obstacles to the integration identified in previous studies. The findings highlight the role of effectively implemented, organised EbyE activity.

## Introduction

In Finland, the utilization of Experts by Experience (EbyE) in mental health services has increased. In addition to the EbyE activity of the non-governmental-sector, effort has been made to expand EbyE activity in the public sector, with the aim of providing client-oriented services and implementing a mental health policy that emphasises service users’ participation and rights at both national (Ministry of Social Affairs & Health, 2020) and international level (World Health Organization, 2021). This qualitative study examines the integration of EbyE activity into public mental health services in one Finnish hospital district, where EbyE activity is already an established working model.

The roots of EbyE activity lead to voluntary peer support, but, in Finland for example, expertise by experience and peer support are seen as two distinct concepts. *Peer support* is still seen as largely voluntary by nature. *Expertise by experience*, on the other hand, is seen as training-based, remunerated activity, which increasingly and in addition to peer-to-peer support, takes place alongside professional practice and is aimed at service development. (Rissanen, 2015, p. 194; Laitila, 2019.) In Finnish context, *EbyE activity* with strong structures has been referred to as *organised experiential expertise* (Hokkanen et al., 2017).

On the other hand, the reality is the overlap between the roles and concepts (see, e.g. Barker & Maguire, 2017). *Peer support workers* also work as paid employees in public sector organisations (e.g. Wall et al., 2021), and *peer support work* can also be based on long-term training and implemented in professional settings such as hospitals or mental health care teams (Nossek et al., 2021; Otte et al., 2020). In addition, peer support workers work with individuals as well as with groups, in psychiatric wards, advocacy, etc. (Moran, 2017, p. 350). As the concepts and practices tend to overlap, and because peer support work has received more attention than EbyE activity in international mental health research, especially when discussing the integration of the activities, this study focusing on EbyE activity also refers to research on (paid) peer support work.

Both peer support work and EbyE activity are seen as beneficial in mental health services. They can improve the well-being of clients, empower peer support workers and EbyE actors themselves, renew the organisation’s ways of working, improve the quality of services and have positive economic impacts (e.g. Gates & Akabas, 2007; Kivistö et al., 2021; Mulvale et al., 2019; Rissanen, 2015), although more research evidence is needed especially on actual mechanisms (Bellamy et al., 2017) and economic impacts (Wall et al., 2021). On the other hand, the implementation of peer support work and EbyE activities in the public sector and traditional professional settings has been considered challenging, and the degree of integration in mental health services varies between organisations (e.g. Bellamy et al., 2017; Gates & Akabas, 2007; Haapakoski et al., 2018; Ibrahim et al., 2020; Moran, 2017; Mulvale et al., 2019; Repper & Carter, 2011). In Finland, there has also been criticism regarding the risk of EbyE becoming restricted to fit a mould dictated by the service system (Meriluoto, 2018; Palukka et al., 2019; Rissanen, 2015, p. 209). Internationally, the same debate has taken place regarding peer support workers (Bellamy et al., 2017).

International research on mental health peer support work has been carried out from the perspective of both integration and implementation. According to Mancini (2018, pp. 127–128), overcoming the challenges related to integration, e.g. solving any questions of authority between experiential and professional expertise, is a prerequisite to effective implementation. In this study, successful implementation is seen as a crucial part of the integration (see Gates & Akabas, 2007, p. 302; Otte et al., 2020). *Implementation* is defined as the deployment and execution of a new operating model, and *integration* is understood as the goal of merging previously separated elements (or operating models) into a more comprehensive whole as a means of improving service quality and efficiency (Finnish Thesaurus and Ontology Service; Kodner & Spreeuwenberg, 2002, pp. 1–2). In Finland the current goal is to integrate EbyE activity into mental health care through systematic implementation.

Based on international research, integrating peer support work into conventional mental health settings poses challenges at both the individual and organisational levels (Moran, 2017, p. 347). Challenges related to the working environment, tasks and personal mental health of peer support workers have been identified (Moran et al., 2013). On the other hand, peer support workers’ work satisfaction, the clarity of roles and responsibilities, the possibility of autonomy and perceptions of inclusion and acceptance within the organisation have been identified as facilitators. Previous research has conceptualised peer support workers’ self-reported, optimal, recovery-oriented work-role model, including three interrelated domains: skills (sharing their lived experience, role modelling, listening and helping clients), relationships (with peer clients, professionals and other peer support workers) and motivations (sense of autonomy, competence and relatedness, occupational commitment) (Moran, 2017). Professionals have also emphasised the importance of clear roles, but in addition, they attach importance to the flexibility of their professional boundaries and perceptions, cooperation and support given to peer support workers (Mancini, 2018, pp. 129–135; also Gates & Akabas, 2007; Hornik-Lurie et al., 2018; Kuhn et al., 2015; Otte et al., 2020; Wall et al., 2021). Professionals have experienced peers offering new perspectives and a positive impact on treatment, care and rehabilitation. However, they have also experienced peer support workers risking their own mental health instead of helping patients, at least in acute phases. (Hornik-Lurie et al., 2018, p. 576.) Some professionals have been unenthusiastic about the utilization of peers, and not all have recognised the benefits of peer support work. Some professionals have set excessive requirements for mental health peer support workers, or they continue to view them as patients. (Gates & Akabas, 2007, pp. 297–306.) There have been calls for cooperation between professionals and peer support workers to remove the obstacles to integration (Vandewille et al., 2016). Factors related to the individual organisation have also been found relevant (e.g. Otte et al., 2020). It is recognised that the implementation and integration of peer support work require an organisational culture that supports the activities, the willingness of the organisation and management, and equal treatment of peer support workers. In addition, support structures such as resources, programmes, training of staff and peer support workers, a clear definition of the roles of different actors, and supervision and management of the activities have been highlighted as important. (Ibrahim et al., 2020; Mancini, 2018; Mulvale et al., 2019; Nossek et al., 2021; Repper & Carter, 2011.)

In Finland, EbyE actors have been identified to have both hierarchical and partnership-based positions in relation to professionals (Soronen, 2021). It has been noted that increasing EbyE activity in the public sector requires good integration of experiential expertise and professional expertise (Palukka et al., 2019; also Jurvansuu & Rissanen, 2018), a change in the working culture, and coordination and support for the activities (Hirschovits-Gertz et al., 2019), such as providing work supervision to EbyE (Jurvansuu & Rissanen, 2018). For example, there have been challenges in providing access for EbyE to the hospital organisation, although once there, they can have a recognised role (Haapakoski et al., 2018). EbyE activity has been seen as a client-oriented activity that complements other services, but its introduction can be impeded by prejudice and lack of knowledge about the competencies of EbyE actors and the purpose of the activities (Hämeenaho et al., 2019). Trust, professionals’ capabilities for cooperation, and physical facilities are identified as facilitators of successful cooperation between professionals and EbyE (Kirjavainen & Hietala, 2019, pp. 252–255).

Previous research has examined the integration of peer support workers into the work community of mental health service settings in the public sector (Kuhn et al., 2015), in mental health care teams (Otte et al., 2020) and mental health clinical settings (Mulvale et al., 2019), in multidisciplinary teams in psychiatric hospitals (Nossek et al., 2021) and as part of the personnel of mental health agencies (Gates & Akabas, 2007). Integration of EbyE acitivity to the hospital organisation has also been studied (Haapakoski et al., 2018). This study complements previous research, focusing on the integration of *established* EbyE activity of one hospital district in Finland. Therefore the study also has the potential to produce the sought-for information on *how* to successfully overcome the challenges of integration (see e.g. Wall et al., 2021; cf. Mancini, 2018; Mulvale et al., 2019; Nossek et al., 2021; Otte et al., 2020). The aim of the study is to describe the experiences of mental health work professionals and trained EbyE actors in integrating EbyE activity into mental health services, and to identify factors that influence the integration. The research question is: *What kinds of facilitators and challenges are associated with integrating EbyE activities into mental health services in the public sector?*

## Methods

### Context

Mental health and substance abuse services are incorporated together in Finland. The responsibility of the public sector for organising mental health services is divided between municipalities^1^ and hospital districts. Mental health and substance abuse services in hospital districts are part of psychiatric specialised medical care. The EbyE activity in the mental health services of the hospital district, which is the context of this study, is wide-ranging and well established. It has developed for more than ten years based on a client-oriented *recovery orientation* in which equality, lived experiences and peer support are valued (see, e.g. Anthony, 1993; Hornik-Lurie et al., 2018; Moran, 2017; Roberts & Boardman, 2014). Paid EbyE activity in the hospital district is based on one year of training and involves experience sharing and personal recovery stories, as well as performing competence-based tasks alongside professionals (see Hirschovits-Gertz et al., 2019). The hospital district signs employment contracts with EbyE and currently employs more than 60 trained EbyEs who perform close to 3000 assignments each year. The tasks of EbyE activity include, for example, lectures, EbyE panels, peer and support roles, group guidance and participation in service development. It can be reasonably said that EbyE activity has been implemented in the hospital district.

### Participants

The hospital district contact person shared the invitation to participate in the study. The only criterion for participation was at least six months of experience working with EbyE actors (for professionals), or some work in an EbyE role in the past six months (for EbyE actors). Professionals were able to participate during working hours, and EbyE actors were offered compensation for any travel expenses incurred as a result of participating in the study. Professionals came from various units of the hospital district, and they included nurses, head nurses, mental health nurses and psychologists. Most of them worked in clinical settings, but some were involved in the training of experts by experience or in the development of EbyE activities. Some professionals had little experience working with EbyE, but many had several years of experience. The average work experience of EbyE actors was a little over 5 years, ranging from 2 to 10 years. EbyEs had lived experiences with mental health problems, substance abuse or eating disorders. They had extensive experience with various tasks. Almost everyone had experience in lecturing, peer support for individuals, working in wards, service development, and guiding groups alone or with a professional. The EbyEs worked not only in clinical and therapeutic groups, e.g. anxiety management group, groups in substance abuse treatment, bipolar support group, or DBT group (dialectical behaviour therapy), but also in leisure groups such as literature group or exercising group. Development work included participation in the development of care and in the design of new facilities. Some had also worked in EbyE training.

### Data Collection

The separate professional and EbyE actor focus group interviews were conducted on the hospital district’s premises in June 2020. We followed the principle of homogeneity (Morgan, 1997) when focusing the groups, as we wanted to ensure that the views of each different *group* related to EbyE activity became visible in relation to the research focus, the facilitators and challenges of integrating EbyE activity. Focus groups are considered a cost-effective method of data collection that provides interactive information from multiple key persons simultaneously (Fern, 2001; Morgan, 1996, 1998). Ten people registered for the professional focus groups, but one cancelled their participation. Four women participated in the first focus group of professionals, and five women participated in the second. None of the EbyE actors withdrew their participation. The first EbyE actors’ focus group had five participants, and the second one had eight participants. Three EbyE were men and ten were women.

The researchers who conducted the focus groups (professional focus groups Author 1 & EbyE focus groups Author 4) had previous experience in conducting research interviews. The focus group interviews were based on a thematic interview framework designed jointly by the research group (academics, including persons with previous experience working as professionals in mental health or substance abuse practice). The interview frameworks were mostly identical for both professionals and EbyE actors. The interviews did not directly ask how the mental health services of the hospital district had succeeded in integrating EbyE activities, but one of the objectives recorded in the interview framework was to obtain information about the strengths and challenges of EbyE activities of the hospital district and ideas on how to develop the activities. The focus groups had extensive discussions about issues related to the integration of EbyE activities, especially when discussing the themes “experiences of EbyE activities and their different forms”, “EbyE activities alongside professional activities”, and “development needs in EbyE activities”. Audio recordings made from the focus group sessions ran to approximately 1.5–2 h.

### Data Analysis

The audio recordings were transcribed verbatim and anonymised. The transcription produced 103 pages of material from the professionals and 94 pages from the EbyE actors. The analysis method was abductive content analysis, in which the framework and units of analysis are not directly based on theory, instead moving between empirical and theoretical, but familiarity with previous research literature can manifest, for example, in the abstraction of categories (see, Graneheim et al., 2018). Author 1 had lead responsibility for implementing the analysis, but the entire research group familiarised themselves with the data. The formation and interpretation of the categories for the analysis were discussed between Authors 1, 2 and 3. All four Authors approved the analysis and the final manuscript.

In the analysis process, Author 1 first analysed the professional data which she had collected and preliminarily analysed immediately after the focus groups had been implemented. The material was systematically read, and the findings that were interpreted as describing the factors related to the integration of EbyE activity in mental health services were chosen for further analysis and categorization. After this, the EbyE data were read and condensed, and they were examined against the observations and categorisations determined from the professional material. It was observed that the comments of the professionals and EbyE actors about EbyE activities and their integration were remarkably consistent. Observations made on the condensed material were first grouped, from the perspective of the research question, into subcategories describing the facilitators and challenges of integration, and then into more abstract head categories (see Elo & Kyngäs, 2008; Graneheim et al., 2018). Both the professional and the EbyE data highlighted positive experiences of EbyE activity integration in the mental health services of the hospital district. Three head categories and 13 subcategories (Table 1) were named to describe the facilitators, and challenges were grouped into two head categories and eight subcategories (Table 2).

**Table 1:** Facilitators for the integration of EbyE activity

Subcategories	Head gategories	Main category
Management’s commitment (P)	An organisational culture that values EbyE	Client-orientation and willingness to develop
Positive attitudes of professionals (P, EbyE)^a^		
Cooperation between professionals and EbyE (P, EbyE)		
Resourcing of EbyE activities (P, EbyE)		
Understanding of experiential expertise (P, EbyE)		
EbyE training (P, EbyE)	Operating structures that facilitate EbyE activity	
Coordination of EbyE activities (P, EbyE)		
Instructions and clear roles (P, EbyE)		
Support for EbyE actors (P, EbyE)		
Feedback and evaluation (P, EbyE)		
Systematic embedding (P, EbyE)	Extensive implementation and utilization of EbyE activities	
Diverse EbyE actors (P, EbyE)		
Different types of EbyE activity (P, EbyE)		

^a^*P* professionals, *EbyE* Experts by Experience

**Table 2:** Challenges to the integration of EbyE activity

Subcategories	Head categories	Main category
Possible management turnover (P)	Uncertainties related to the different actors	Need for continuous development and reflection
Reserved attitude to EbyE among some staff (P, EbyE)		
EbyE actors’ coping ability (P, EbyE)		
Achieving genuinely equal status for EbyEs (P, EbyE)	Development needs related to operating practices	
Continuous diversification of activities (P, EbyE)		
Visibility and continuous expansion of EbyE activity (P, EbyE)		
Strong guidance (P)		
Obtaining research evidence (P)		

### Ethics

The principles of the responsible conduct of research were followed at all stages of the study (Finnish Advisory Board, 2012). The ethical approval was given 25th March 2020 by the Research Ethics Committee of University of Lapland. A research permit was applied for from the hospital district. The study participants were informed both in advance and in the research setting about the purpose of the study and matters related to data protection and data management. Participation in the study was voluntary, and the anonymity of the participants was protected. Informed consent was obtained from all the participants. For research quality, the research design included the perspective of two different groups of actors, and in order to assess the consistency of the findings and the data, sample tables and extracts are presented (see, Graneheim et al., 2018; Silverman, 2005). The COREQ checklist for qualitative research (Tong et al., 2007) was used to ensure quality where applicable.

## Results

Based on the analysis, the facilitators for integrating EbyE activity into mental health services were identified as follows: *an organisational culture that values EbyE*, *operating structures that facilitate EbyE activity*, and *the extensive implementation and utilization of EbyE activities*. These facilitators were interpreted as reflecting *client-orientation and willingness to develop* in the organisation (see Table 1). In addition to positive views, the data also made visible some challenges that pose certain requirements to the integration of EbyE activity both now and in the future. The challenges of integrating EbyE activity were itemised into the *uncertainties related to the different actors* and *development needs related to operating practices*. The challenges reflected the need for *continuous development and reflection* regarding the activities (see Table 2).

### Organisational Culture That Values EbyE

The experiences of the study participants indicated that an organisational culture in which EbyE activity is valued had provided a good foundation for the integration of EbyE activity in the hospital district’s mental health services. According to the professionals, central to this were actions by the management of the hospital district’s psychiatric domain, which they described as being openly committed to EbyE activities.

*For sure [the name of the medical director of psychiatry] should be given credit for this, for launching it here and being enormously supportive of this issue—. *(P1P2)^2^

Professionals reported that EbyE activities were included in the hospital district’s strategy. They saw EbyE activity as an integral part of the hospital district’s mental health and substance abuse services, and described the role of EbyEs in the services as “self-evident” and “involved in everything”.

*It [EbyE activity] is part of this whole system alongside treatment, it must be and it belongs here.* (P1P1)

Like the professionals, EbyE actors also found that the attitudes of the hospital district personnel towards EbyE had become more positive in recent years.

*In 2015, with nurses it was still a little bit like they saw it [EbyE activity]as a threat, and what would their role be if they [EbyE actors] were there. And back then one doctor had said that if crazy people are treating crazy people, what good will that do? But thankfully that has changed.* (EE1P3)

Professionals who participated in the focus groups had very positive experiences of EbyE activities, and many asserted outright pride in them.

*But I have to say that it is great that we have got this [EbyE activity] so advanced here*. (P2P4)

*Yes, we should be proud, and I’m sure we are*. (P2P1)

Like the professionals, EbyE actors also felt that the situation with EbyE activities was better in their hospital district than in many other regions or localities in Finland.

*[The hospital district] has been a pioneer in these matters—. *(EE2P1)

*When you go outside of this area, it [EbyE activity] becomes more difficult straight away. Here the reception has been good, and they know who we are and how they can use us and where.* (EE2P4)

The study participants were satisfied with the well-working cooperation between professionals and EbyE. Equality, mutual trust and appreciation were seen as the starting points for successful cooperation. EbyE participants of the focus groups felt that they were valued members of the work community.

*It feels like they [professionals] appreciate this experiential expertise enormously and what we have gone through in our own lives—. And coming into the work community, we are treated as equal.* (EE1P5)

The resources allocated to EbyE activities included organising long-term training for the EbyE and paying them for both completed and unexpectedly cancelled assignments. Attention was paid to the workspaces of EbyE actors, and work supervision was available for them. EbyE actors themselves also valued various symbols related to the work, such as name tags, keys and passes, which signal that they are valued by the hospital district.

*We belong somewhere. We can walk around in the hospital, we have the same passes [electric keys] as everyone else.* (EE1P1)

In the data, the existence of an organisational culture that values EbyE activity was also linked to understanding the nature of experiential expertise. While professionals emphasised the benefits of EbyE activity for clients and the service system, on the other hand they emphasised the non-professional nature of the activity. It was pointed out that EbyE should not be overloaded with too much work. EbyE actors also reported that in the hospital district it is understood that an EbyE actor’s own recovery can sometimes take a setback, and it can be reflected in the performance of their tasks.

### Operating Structures That Facilitate EbyE Activity

The hospital district had established operating structures for EbyE activity, which included EbyE training and centralised coordination of EbyE, including assignment allocation. The hospital district has a full-time EbyE coordinator and a core working group for the coordination, training and development of EbyE activity. Both professionals and EbyE actors emphasised the importance of long-term and versatile EbyE training, including lectures, group meetings and presentation training. The 1-year training organised in-house by the hospital district was also mentioned as a key reason for the positive attitudes towards EbyE activity.

Professionals saw importance in the ease utilization of EbyE actors, which was related to centralised coordination in the hospital district. If necessary, the EbyE coordinator provided assistance in finding a suitable EbyE actor, but information about EbyEs, their areas of expertise, and instructions on how to “book” an EbyE were also available to professionals in the information system and manual folders.

*—and we have instructions there, like folders, where you can look, and the booking of an expert by experience and clear instructions, so even new ones [employees] can look there. So if you don’t know how they can be booked, you can read and learn.* (P1P1)

Professionals reported that the job descriptions of EbyE actors were approved by the hospital district’s management team, and EbyE actors were told about their future tasks, roles and responsibilities already during the training. The tasks were also reviewed before they were started. Both professionals and EbyE actors emphasised that, when using EbyE as part of the care, the responsibility for the activity always rests with professionals, and they considered the EbyE instructions and clear roles to be important.

*—they [a professional] kind of bring structure to the group and the content, the topic in each case, and then the expert by experience provides their own service, their own experience of the topic at hand—the patients are aware that an expert by experience is involved in their care.* (P1P2)

*It [a specific treatment model] is highly structured and there are topics that are taught, so the professional brings the so-called theoretical side, and my task is to bring the practical side, how I have learned it, how it shows in my everyday life and so forth.* (EE2P6)

The professionals emphasised the meaningful role of EbyE actors in mental health services and in supporting clients, instead of “making coffee”, “opening a door”, “taking rubbish out” or “washing dishes”, and stated that detailed job descriptions not only define the role of EbyE actors in relation to the tasks of professionals, but they also prevent EbyE actors from being “pushed around”.

Operating structures that facilitate EbyE activity included support for EbyE actors in the form of work supervision and development discussions. In addition, peer support networks had been established for and by EbyE actors. The collection of multi-directional feedback and regular evaluation of the activities had also become established practices in EbyE. Both professionals and EbyE actors emphasised the importance of both positive and critical feedback to facilitate development.

### Extensive Implementation and Utilization of EbyE Activities

According to the study participants’ experience, an organisational culture in which EbyE activity is valued, and the facilitating operating structures, had contributed to the extensive adoption of EbyE in the mental health services of the hospital district. The current EbyE activities were described as well-established, but it was emphasised that the implementation had not been instantaneous and had required systematic embedding.

*And now that it’s [EbyE activity] well established, it has a different grounding from which to progress*. (P2P5)

*A path has been cleared for us.* (EE2P3)

The EbyE activities organised in the hospital district’s mental health services were versatile. Patients were offered a wide range of support by EbyE actors. EbyE actors were utilized in both rehabilitative and acute phases and in both individual and group activities. Trained EbyE actors worked in the hospital district both independently and in cooperation with professionals. EbyE actors were also involved in project activities, development, induction training for new employees such as doctors, and as members of the hospital district’s management team. Some EbyE actors visited wards to talk to patients or give lectures about their own experiences. Some were paired with professionals and worked in clinical settings. One of the professional participants had started developing a new treatment programme in cooperation with an EbyE and a patient who was attending EbyE training.

*We are currently developing, to go alongside this standard DBT [dialectical behaviour therapy], another application—a treatment programme—. We are, me and the expert by experience—we did the development work together and are taking it forward. And then for the pilot we took a patient—who—is now in expert by experience training. So as a group, me as a professional, the expert by experience and a patient who is now undergoing a metamorphosis from a patient into an expert by experience, this group of ours is now developing these practices.* (P2P3)

Versatile EbyE activities also ensured that EbyE actors were given tasks that were suitable for them individually. For example, one of the EbyE did not feel that sharing their own experiences would suit them. Based on the EbyE actors’ descriptions, the EbyE training organised by the hospital district had enabled them to act as EbyE according their own individual strengths.

*There is perhaps the point that we are not educated [in the EbyE training] according to a standard formula, but everyone becomes—everyone has their own strength, so one person focuses on this, another one on that, and then the instructors [professionals] can pick the right help provider.* (EE2P3)

### Actor-Related Uncertainties in the Integration of EbyE Activity

Although the experiences of study participants emphasised successful integration of EbyE activity in the hospital district’s mental health services, some concerns were expressed. Uncertainties in EbyE activities were linked to different human actors: managers, personnel and EbyE actors. For example, as a flip side to the finding that the current management of the psychiatric domain was seen committed to the EbyE activities, some professionals wondered whether the activities would be at risk if there were changes to the management. On the other hand, they believed that the commitment was now well-established.

*Our previous head nurse—was strongly in favour of these expert by experience activities, and I believe that the new head nurse also supports it.* (P1P4)

The focus groups suggested that even now, there are some professionals who do not see the benefits of EbyE. Criticism from the EbyE actors mainly focused on external professionals outside the hospital district, but professionals also reported that some of the staff in their hospital district still had reserved attitudes or an “ossified” tendency to stigmatise all EbyE actors based on one negative experience.

*It’s sad that some individuals [employees] tend to stigmatise all the good—. *(P2P5)

Some felt that professionals were too keen to see problems in EbyE activities.

*Patients and experts by experience don’t see problems where we, the staff, might see problems—. *(P1P2)

Professionals who participated in the focus groups emphasised that any negative experiences related to EbyE actors were individual and exceptional cases. Challenges regarding the EbyE actors were more likely to relate to their own coping, as the number of assignments performed by EbyE actors had constantly increased in the hospital district. On the other hand, some professionals strongly questioned why the coping of experts by experience specifically should be monitored—after all, professionals can also be tired and even unable to work at times.

*On the other hand, what determines that, if someone else determines and says okay, now you [EbyE actor] are in such a state that you can no longer perform, you are more like a patient now. So it’s challenging.—But then I wonder, why the expert by experience, why not any colleague [professional], if you see that they are not well?* (P2P3)

The EbyE actors’ focus groups also discussed coping and the need to limit their tasks. It had been difficult to refuse offered assignments, because serving as an EbyE actor was seen as meaningful and purposeful.

*The challenge is your own coping, and for me the most difficult challenge is setting limits. I take on so many tasks, and then the exhaustion—. It would be so great to take part in everything—. *(EE1P4)

With further experience and mutual support, EbyE actors had learned to limit their work tasks. However, it was also reported that some had experienced burn-out.

### Development Needs Related to Operating Practices of EbyE Activities

The study participants reported certain development needs related to practices of EbyE activity. For example, both professionals and EbyE actors reported that EbyE were not in a position to access all work tools, such as computers and online access, on a fully equal footing with professionals. EbyE actors expressed wishes for a work phone, the possibility to work more full-time, trade union membership and access to occupational health care. Not all EbyE actors had heard about the possibility of work supervision. Some EbyE actors felt that the current remuneration was too low, and some had encountered difficulties in balancing their pay and social security benefits. Those who had worked as EbyE actors for a long time called for the possibility of updating their EbyE profile in the hospital district information system as well as additional or continuing education.

*I think that additional training should be developed, because in [current] practice there is none.* (EE1P4)

Despite well-working cooperation between professionals and EbyE actors, the difficulty of achieving genuine equality or even defining what it would be, was reported in the data. Although the tasks and roles of different actors were clarified, it was felt that the boundaries were still partly fluctuating between professionals and EbyE actors as well as between EbyE actors and patients. Some professionals wanted more instructions for challenging situations. EbyE actors wanted more interaction with the staff, for example at wards, so that the staff would have a more accurate idea of the type of EbyE actors needed in each assignment. They also wanted more detailed induction.

*The request that comes [to the EbyE actor], about a task, it’s a very short description about what the author of the order wants or what the client wants? So when you go and do the task, it would be good to have a briefing from the staff, what they want and—. What is the approach that they are looking for?* (EE1P5)

With regard to evaluation of EbyE activities, EbyE actors stated that their opinions were asked, but they wished that the feedback would be shared more and lead to more concrete measures.

Although the hospital district already had versatile EbyE activities, the study participants wanted to see more. For example, professionals suggested the development and introduction of EbyE activities working with somatics, families with children and on an on-call basis, and the use of equipment such as telephone or online services. EbyE actors suggested that they could be systematically involved in the early stages of the care processes and in multidisciplinary teams and all recruitment.

Professionals said that they were keen to use EbyE actors who showed initiative, although “quiet” ones were also appreciated. They hoped to see more men get involved. Personal chemistry was considered significant both between professionals and EbyE actors and between clients and EbyE. According to professionals, involving different persons in EbyE activities should be a consideration already during the recruitment of participants for EbyE training. Both professionals and EbyE actors stressed that EbyE should be used equally and rapid access to the activities should be ensured for newly graduated EbyE actors.

*We perhaps tend to use the same people [EbyE actors] a lot of the time, so if we could have a bit more variety.* (P2P1)

*Because there are new ones [EbyE actors] graduating all the time, so it would be good to give them a chance*. (EE1P1)

Both professionals and EbyE actors considered it important that EbyE activity is discussed publicly and made more visible. They also hoped that the activity could be expanded nationally more than it currently is.

*I would hope that this kind of expertise by experience would be used in other hospital districts as well.* (P2P4)

The professionals highlighted the need to strengthen the guidance on EbyE activities so that there would be an obligation to trial EbyE in all types of groups and wards of the hospital district, but also in other public sector services nationwide. The professionals also stated that EbyE activity would benefit from research that makes its impacts visible. Professionals who participated in the focus groups had a strong impression of the benefits of EbyE activity, but they recognised that the implementation of new operating models and practices in mental health services requires research evidence.

*I think at least doctors look at that, and if there is no research evidence, then it does not exist*. (P1P2)

## Discussion

The study presented the facilitators and challenges of integrating EbyE activity in mental health services in one Finnish hospital district, where EbyE activity is a wide-ranging and established operating model. Based on the results, the starting point for integration is an organisational culture in which expertise by experience is understood and EbyE activity is valued by the management and staff throughout the organisation as comprehensively as possible. Integration requires operating structures that facilitate and support the EbyE activity, such as training of EbyE actors and centralised coordination of the activities. Integration also requires extensive implementation and utilization of EbyE activities. Together, these facilitators can be interpreted as a model for integration of EbyE activity.

Experiences of the successful integration of EbyE activity were emphasised in the study, and this was interpreted to reflect the hospital district’s client-orientation and willingness to develop. However, the professionals and EbyE actors also mentioned certain integration challenges related to actors and practices, which were interpreted to reflect a need for continuous development and reflective assessment of the EbyE activities. Uncertainty was felt in relation to possible changes in the management of the hospital district’s psychiatric domain and the potential impacts, negative attitudes among some professionals, and EbyE actors’ ability to cope. Development needs relating to practices appeared to include, for example, the attainment of genuine equality for EbyE actors in the work community, continuous diversification of activities, and obtaining research evidence about the benefits of EbyE.

The mental health professionals and EbyE actors who participated in the study described the integration of EbyE activities in the hospital district in a consistent manner. The main differences were that, unlike EbyE, professionals also identified management, guidance and research evidence as important factors (see Table 1 and Table 2). EbyE actors, on the other hand, were more likely to emphasise concrete benefits, facilities and symbols related to equal and valued status in the work community. These findings can be interpreted as indications that professionals place particular importance on factors which are related to the organisation and management of EbyE activity, while EbyE actors place importance on factors that are related to their own professionalism.

Overall, the findings of this study align with earlier research on the facilitators and challenges of the integration of peer support work and EbyE activity in traditional mental health services. However, the participants’ experiences of integration were more positive in this study compared to most earlier studies (c.f. e.g. Hämeenaho et al., 2019; Mulvale et al., 2019; Nossek et al., 2021; Otte et al., 2020; Vandewille et al., 2016; Wall et al., 2021). Positive attitudes, the role clarity, supportive policies and practices, and concrete support (see Gates & Akabas, 2007) were highlighted as realised facilitators in this study. For example, the lack of clarity of roles has often been identified as a key challenge for integration (e.g. Gates & Akabas, 2007; Ibrahim et al., 2020; Mancini, 2018; Otte et al., 2020). On the other hand, it has been noted that the roles become clearer over time and as the integration progresses (Nossek et al., 2021). The findings of this study support that observation, as the development of EbyE activity had started in the hospital district more than ten years earlier.

The study findings indicate that the hospital district had strived for ensure that employees were informed about the skills of EbyE actors and the objectives of the EbyE activity (cf. Hämeenaho et al., 2019), and that the actors and job tasks were coordinated (cf. Hirschovits-Gertz et al., 2019). Centralised coordination of EbyE activities in the hospital district can be interpreted to respond to the previously identified need for organisation and management (see, e.g. Gates & Akabas, 2007; Hirschovits-Gertz et al., 2019). As EbyE activity was an established working model in the hospital district, the findings also emphasised the importance of systematic implementation and the role of activities in the realisation of a client-oriented approach in the organisation (see, Mancini, 2018). The latter observation may also be related to the hospital district’s commitment to recovery orientation (also Gates & Akabas, 2007), which has been identified as a meaningful premise for integration of peer support work (Hornik-Lurie et al., 2018; Moran, 2017).

Findings regarding the integration of EbyE activity confirm previous observations on peer support work, namely, that the implementation of the activities in mental health services requires extensive organisational readiness and determination (Mancini, 2018, p. 136). The findings indicate that the integration of EbyE activities into mental health services in the public sector can be achieved in a managed and coordinated manner, and recognised as an organised activity. Organised, training-based, structured and formalised EbyE activities can strengthen the continuity and credibility of the activities and make them more flexible and resilient to potential changes in resources, actors or conditions (Hokkanen et al., 2017, pp. 1156–1157; see, Martin et al., 2021; Wall et al., 2021).

On the other hand, organised and established EbyE activities also require continuous evaluation. In the study, some questions remain unanswered, e.g. the degree of professionalism of EbyE actors (also Otte et al., 2020) and tensions regarding how and to what extent EbyE activities, which originate from notions of parity, mutuality and peer volunteering, are organised and managed through the public sector service system (also Kivistö et al., 2021). As Kuhn et al., (2015, p. 453) state, it is important to maintain the uniquely peer role at the same time as becoming an integral part of the services. In this study, despite successful integration, equal access for EbyE actors to all resources of the organisation and the attainment of certain factors related to equal working-life status appeared to be a challenge (also Gates & Akabas, 2007; Ibrahim et al., 2020; Mancini, 2018; Nossek et al., 2021; Wall et al., 2021). Also in alignment with previous research, the coping of EbyE actors was considered a challenge; however, some professionals questioned the necessity of monitor it (Gates & Akabas, 2007; Otte et al., 2020).

Regarding the concept of integration, it is necessary to critically evaluate, whether EbyE activity is integrated into traditional mental health services in public sector as a “top-down” process (Kodner & Spreeuwenberg, 2002, p. 5; see, Bellamy et al., 2017). In this study, integration appeared to mean both horizontal and vertical integration. For example, the role of the hospital district’s management and qualified professionals was emphasized, but so was the cooperation, reciprocity and equality of all actors, as well as the importance of lived experiences alongside professional mental health protocol (also Mancini, 2018; Moran, 2017).

Based on this study, the hospital district has overcome many of the challenges identified in previous studies on the integration of peer support work and EbyE activity in mental health services. Overall, the findings highlight the role of effective implementation in integration. The findings can be interpreted as evidence of *organised experiential expertise* (Hokkanen et al., 2017) as a general facilitator for integration.

If the study had been initiated as a longitudinal research design at the beginning of the development and implementation of EbyE activity in the hospital district, it would reflect integration as a process more specifically. Furthermore, a limitation of the study, was the small number of study participants, who may have had particularly positive views about EbyE activity in the hospital district. The context of the study was case-specific, however, the challenges of integration have been identified regardless of the sociocultural context or psychiatric setting (Otte et al., 2020, p. 267). The facilitators and challenges identified in the study can be found in other mental health facilities in the Western world, even if not exactly identical. This study contributes to the goal of integrating EbyE activity into mental health services in accordance with current national and international mental health policies.

## Footnotes

1 Wellbeing services counties from 2023.

2 The anonymised extracts that demonstrate the connection between the findings and the data are identified by reference to the focus group and the interviewee (e.g. P1P4 means participant four of professional focus group 1, and EE2P3 means participant three of EbyE focus group 2). Some redundant filler words were removed from the extracts. Text sections that were not relevant for interpretation are marked with dashes—. Clarifications are presented inside square brackets [].
